# Causes of and Molecular Targets for the Treatment of Intervertebral Disc Degeneration: A Review

**DOI:** 10.3390/cells11030394

**Published:** 2022-01-24

**Authors:** Takashi Ohnishi, Norimasa Iwasaki, Hideki Sudo

**Affiliations:** 1 Department of Orthopedic Surgery, Hokkaido University Hospital, Sapporo 060-8648, Japan; takashi.onishi.ortho@gmail.com; 2 Department of Orthopedic Surgery, Faculty of Medicine and Graduate School of Medicine, Hokkaido University, Sapporo 060-8638, Japan; niwasaki@med.hokudai.ac.jp; 3 Department of Advanced Medicine for Spine and Spinal Cord Disorders, Faculty of Medicine and Graduate School of Medicine, Hokkaido University, Sapporo 060-8638, Japan

**Keywords:** intervertebral disc degeneration, apoptosis, necroptosis, pyroptosis, ferroptosis, small interfering RNA (siRNA), microRNA (miR), long non-coding RNA (lncRNA), circular RNA (circRNA)

## Abstract

Intervertebral disc degeneration (IVDD) is a pathological condition that can lead to intractable back pain or secondary neurological deficits. There is no fundamental cure for this condition, and current treatments focus on alleviating symptoms indirectly. Numerous studies have been performed to date, and the major strategy for all treatments of IVDD is to prevent cell loss due to programmed or regulated cell death. Accumulating evidence suggests that several types of cell death other than apoptosis, including necroptosis, pyroptosis, and ferroptosis, are also involved in IVDD. In this study, we discuss the molecular pathway of each type of cell death and review the literature that has identified their role in IVDD. We also summarize the recent advances in targeted therapy at the RNA level, including RNA modulations through RNA interference and regulation of non-coding RNAs, for preventing cell death and subsequent IVDD. Therefore, we review the causes and possible therapeutic targets for RNA intervention and discuss the future direction of this research field.

## 1. Introduction

Intervertebral discs are located between the vertebrae and confer mobility, load absorbability, and support to the spinal unit [1]. Intervertebral disc degeneration (IVDD) is associated with discogenic low back pain and neurological complications, imposing taxing health problems and a huge economic burden on humans [2,3]. Particularly, the prevalence of low back pain is markedly high worldwide [4]. The mechanism through which IVDD contributes to low back pain is not fully understood; however, several studies have reported the role of aggrecans in nerve and/or blood vessel ingrowth that can be attributed to the generation of nociceptive and neuropathic pain signals. Stefanakis, et al. suggested that the depletion of proteoglycans reduces the internal hydrostatic pressure of the disc and subsequently facilitates blood vessel and nerve ingrowth [5]. In addition, Johnson et al. revealed that chondroitin sulfates of aggrecan inhibit nerve growth. They also stated that the turnover of these proteoglycans to those that possess more keratan sulfates may promote nerve ingrowth to the disc [6]. Recently, Krock et al. reported that degenerating and painful human IVDs release increased levels of nerve growth factors (NGFs), which are inflammatory and nociceptive factors that may induce neo-innervation and pain [7]. Accordingly, IVDD is considered a factor that can induce low back pain.

Currently available treatments include surgeries, such as discectomy and spinal fusion, or medications to alleviate pain [8,9,10]. The primary problem with these treatments is that they do not address the fundamental underlying pathological conditions but aim to alleviate the symptoms indirectly [8,9,10]. In addition, their efficacy is not always sufficient and can cause adverse side effects, such as relapse of herniation of the nucleus pulposus (NP) [11,12,13], adjacent segment disease [14], or surgical site infection [15]. Therefore, more direct and effective treatments are needed.

The molecular mechanism underlying IVDD includes DNA replication error, metabolic disorder, and inflammation, and the consequent loss of the disc matrix, functional cells, and stem cells is a characteristic of degenerated discs. DNA replication error occurs secondary to DNA damage response, which leads to cellular senescence [16]. The p53-p21-retinoblastoma (RB) protein pathway and p16-RB protein pathway become activated by DNA damage, and this leads to the arrest of cell cycle transition [16,17]. Accumulation of senescent cells invokes senescence-associated secretory phenotype that exhibits chronic inflammation and induces degradation of the disc matrix [18,19]. Moreover, NP cells are metabolically adapted to a hypoxic and low glucose environment; therefore, they rely on anaerobic glycolysis to generate energy with low reactive oxygen species (ROS) production [16,20]. High glucose levels in diabetic patients are known to cause high oxidative stress and induce IVDD [16,21]. Metabolic disorder can also be induced by advanced glycation end product (AGE) accumulation that is associated with IVDD [22]. Degradation and reduction of aggrecan leads to loss of hydration and internal hydrostatic pressure in the NP [23,24]. Inflammation leads to aggrecan loss in the NP, and the involvement of interleukin (IL)-1β [25], IL-6 [26,27], IL-17 [28], tumor necrosis factor (TNF)-α [29], and interferon-γ [28] in this process has been reported. Among the factors that contribute to IVDD, many of them, including metabolic disorder and inflammation, commonly cause cell death.

Involvement of autophagy in the IVD has been also studied, and this process was found to be a double-edged sword in the development of IVDD, depending on the stimuli [30,31,32]. Autophagy induced by oxidative stress promoted apoptosis of NP cells [32], and mechanical stress-induced autophagy triggered apoptosis of annulus fibrosus cells [33]. Conversely, autophagy stimulated by hypoxia or metformin exhibited a protective effect against apoptosis of NP cells [31,34]. These previous studies underscore the complex role of autophagy in the development of IVDD, indicating that the regulation of autophagy may be an approach for IVDD treatments.

Numerous studies have been performed to develop novel treatments for IVDD in stages from the bench level to translational research. Biomaterials and cell-based regenerative treatments are attractive tools for reconstituting terminally degenerate matrices to restore function and mitigate pain. However, early diagnosis and minimally invasive treatments are ideal in the initial stage of the disease, and iatrogenic remodeling of the tissue should be preceded by some intervention to prevent the degenerative processes. Therefore, several treatments are based on the strategy of regulating programmed cell death (PCD) [35] and other types of regulated cell death (RCD) [36] (combined as PRCD), as well as targeting autophagy, in the vicinity of intervertebral discs. Especially, evidence of PRCD in IVDD has been reported by several researchers ever since 2000 [37,38,39,40,41,42]. The loss of local cells is attributed to the attenuated generation of matrix components, such as aggrecan and type II collagen, accompanied by the loss of water aggregated to hyaluronic acid of aggrecan. The reduction of these components coincides with the replacement of the matrix by other fibers, such as type I collagen and fibronectin [43], and other proteoglycans, such as decorin, biglycan, and fibromodulin [44], which results in the formation of a matrix with suboptimal function. To block the initiation of matrix remodeling, presumably local cells of the discs must be maintained. Therapeutic targets to inhibit PRCD have been extensively explored for decades, and this therapeutic principle has become a major strategy to treat IVDD in the early to middle stages [31,45,46]. An emerging and actively explored approach in treating IVDD is RNA targeting, including mRNAs and non-coding RNAs (ncRNAs). In this study, we review the molecular signaling pathways of PRCD and molecular targets to suppress these types of cell death, focusing on mRNA interference and modulation of ncRNAs, thereby inhibiting the progression of IVDD.

## 2. Signaling Pathways and Factors Inducing PRCD in Disc Degeneration

Several types of cell death have been reported to be involved in the occurrence of IVDD. The most well-known PRCD is apoptosis, which is typically triggered by death ligand-receptor binding [47], endoplasmic reticulum (ER) stress [48], and mitochondrial dysfunction secondary to inflammation [49,50], mechanical stress [42], and oxidative stress [51,52]. Other PRCD mechanisms include necroptosis [53,54,55,56], pyroptosis [55,57,58,59,60], and ferroptosis [61,62,63]. Historically, apoptosis has been termed PCD, while the others have been termed RCD [35,36]. Each type of PRCD is considered to contribute to the development of IVDD depending on the underlying pathogenesis. In this section, we explain the signaling pathways for each type of PRCD before introducing the therapeutic targets in the next section.

### 2.1. Apoptosis

**Figure 1:**
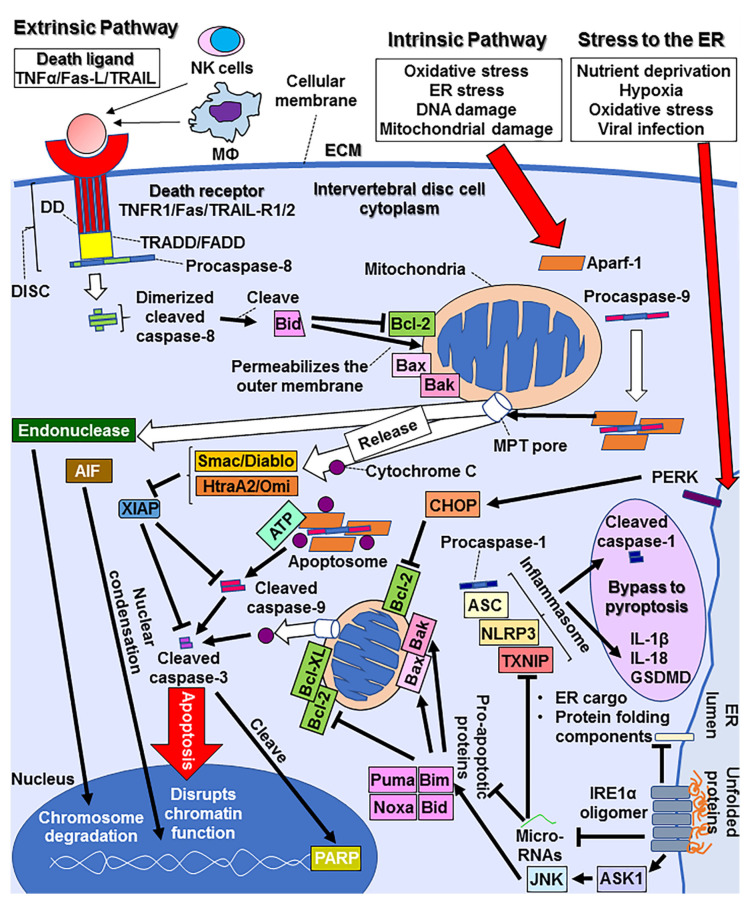
A scheme of apoptotic signaling pathways. AIF, apoptosis-inducing factor; Apaf-1, apoptotic protease activating factor-1; ASC, apoptosis-associated speck-like protein; ASK1, apoptosis-signal-regulating kinase 1; CHOP, C/EBP homologous protein; DD, death domain; DISC, death-inducing signal complex; ECM, extracellular matrix; ER, endoplasmic reticulum; FADD, Fas-associated death domain; GSDMD, gasdermin-D; IRE1α, inositol-requiring enzyme 1α; JNK, c-Jun NH2-terminal kinase; NLRP3, nucleotide-binding oligomerization domain (NOD)-like receptors family pyrin domain containing 3; PARP, poly (ADP-ribose) polymerase; PERK, pancreatic endoplasmic reticulum kinase; TNFR, TNF receptor; TRADD, TNFR-associated death domain; TRAIL, TNF-related apoptosis-inducing ligand; TRAILR, TRAIL receptor; TXNIP, thioredoxin-interacting protein; XIAP, X linked inhibitors of apoptosis protein.

There are two types of apoptotic signaling pathways—intrinsic and extrinsic pathways [42]. The intrinsic pathway is triggered by cellular stresses such as oxidative stress [51,52], ER stress [48], DNA damage [64], and mitochondrial damage [42]. Following these stimuli, an initiator caspase, caspase-9, binds apoptotic protease activating factor-1 (Apaf-1) to induce a decline in mitochondrial membrane potential and opening of the mitochondrial permeability transition pore (MPTP) [35]. This leads to the release of mitochondrial cytochrome c, second mitochondria-activator of caspase (Smac/Diablo), high temperature requirement protein A2 (HtrA2)/Omi, apoptosis-inducing factor (AIF), and endonuclease [42]. Cytochrome c forms a complex with Apaf-1, procaspase-9, and ATP to form an apoptosome [42]. Apoptosomes recruit the active form of caspase-9 to subsequently cleave and activate the effector caspase caspase-3 to induce apoptosis [35]. Caspase-3 cleaves poly (ADP-ribose) polymerase (PARP) and suppresses its effect to deplete ATP [58]. This is attributed to the maintenance of ATP levels in apoptotic cells [58]. Smac/Diablo and HtraA2/Omi are accessory pro-apoptotic proteins that inhibit inhibitors of apoptosis proteins (IAPs) and help initiate apoptosis [35,65]. The release of AIF facilitates nuclear condensation and disrupts chromatin function [42]. Endonuclease activation promotes chromosomal degradation and other processes common between intrinsic and extrinsic pathways, including the destruction of the nuclear proteins and cytoskeleton, crosslinking of proteins, expression of ligands for phagocytic cells, and formation of apoptotic bodies [35]. The extrinsic pathway is initiated by binding of the death ligands and receptors, including TNF and TNF receptor 1 (TNFR1), Fas-L and Fas (alias CD95-ligand and CD95 or APO-1), and TNF-related apoptosis-inducing ligand (TRAIL) and TRAIL receptor 1/2 (alias Apo2-L and TRAIL-R1/2 or DR4) [35]. These ligands are secreted by natural killer cells or macrophages and bind to receptors on the surface of the target cells [35]. The initiator caspase for this pathway is caspase-8. Procaspase-8 interacts with the death domain (DD) that is a cytoplasmic module of death receptor and forms a death-inducing signal complex (DISC), and subsequently cleaves to the active form of caspase-8 [35]. In this process, an adaptor protein, such as Fas-associated death domain (FADD) or TNFR-associated death domain (TRADD) of DISC assists the interaction of procaspase-8 with DISC [35,66]. Dimerized and activated caspase-8 cleaves effector caspase-3 with or without the involvement of Smac/Diablo and HtraA2/Omi inhibition of X linked IAP (XIAP), depending on the cell type [67]. Previous studies have provided evidence of both intrinsic and extrinsic pathways in degenerated disc tissue [37,38,42]. Specifically, cells of the NP have been reported as type II cells, wherein they undergo the extrinsic apoptotic pathway [37]. Briefly, minute caspase-8 expression activates Bid (BH3 interacting domain death agonist) but does not activate caspase-3 directly; Bid heterodimerizing with Bax and Bak, and antagonized Bcl-2 and mitochondrial membrane permeability increases and pro-apoptotic proteins egress to the cytoplasm. The signaling following this is similar to the intrinsic pathway that recruits caspase-9 [37,67] (See Figure 1).

Apoptosis is induced by several factors. One representative model of injury-induced IVDD is the needle puncture model [10,68,69,70]. In a rabbit model of IVDD generated by needle puncture, Fas expression was evident only in the punctured discs, and Fas-L expression was augmented in the punctured discs than in the control discs [71], indicating that injury can cause Fas-L-Fas binding to initiate apoptosis. An increase in the expression of inflammatory cytokines, such as IL-6, TNFα, and IL-1β, has been observed in a rat tail disc puncture model [72]. Considering the existence of TNFR1 and TNFR2 in NP [73], TNFα-TNFR binding can also lead to apoptosis in cells of the NP. Biomechanical overload is a major cause of apoptosis [74,75,76]. In an in vitro compression model of human NP cells, the involvement of the intrinsic pathway of apoptosis, represented by elevated Bax and decreased Bcl-2, was elucidated [77]. Increased levels of oxidative stress result in apoptosis via the intrinsic pathway owing to compression [76]. Because mitochondria are the main source of ROS, compression-induced damage to mitochondria results in the enhanced generation of ROS in the cytoplasm and contributes to increased oxidative stress and apoptosis of NP mesenchymal stem cells via the intrinsic pathway [34,42,76]. The opening of the MPTP is composed of an adenine nucleotide translocator (ANT), cyclophilin-D (CYP-D), and voltage-dependent anion channel (VDAC), which increases the permeability of the mitochondrial membrane [42]. The detrimental outcomes include suppression of the respiratory chain and the release of pro-apoptotic proteins, including cytochrome c [42]. In addition, miRNA-34a-5p is reported to participate in compression-induced degeneration by repressing silent mating type information regulation 2 homolog 1 (SIRT1), which is a critical regulator of cell differentiation, proliferation, and apoptosis [77]. ER stress is another factor that triggers the apoptotic signaling cascade. The basic role of ER stress is to restore homeostasis in the ER and protect cells [33,78]. However, if the stress is excessive or persistent, ER dysfunction may not completely recover, resulting in apoptosis [33,48]. Factors, such as nutrient deprivation, hypoxia, oxidative stress, and viral infection may induce protein misfolding or disarray in calcium homeostasis, leading to ER stress [33,79]. Initially, a signaling pathway called the homeostatic unfolded protein response (UPR) attempts to correct this condition [78,79]. Three ER transmembrane proteins, namely, inositol-requiring enzyme 1α (IRE1α), pancreatic endoplasmic reticulum kinase (PERK), and activating transcription factor 6 (ATF6) sense misfolded proteins at critically high concentrations [48,78,79]. IRE1α and PERK have ER-luminal domains that can dimerize and initiate the UPR; however, in the unstressed state, their binding with the ER chaperone BiP suppresses the UPR [79,80]. Unfolded proteins can titrate BiP and bind to the ER luminal domain to initiate the UPR [48,79]. When homeostatic UPR fails to restore the condition by increasing ER size, chaperone biogenesis, degradation of misfolded proteins, and slowing down protein translation, the signaling platform transforms to a terminal UPR [78,79]. Hyperactivated IRE1α forms an ER-luminal domain oligomer, which allows RNase to degrade mRNAs that are not appropriate substrates, such as ER cargo and protein-folding components, and worsens ER stress [48,79]. In addition, RNase insult leads to a reduction in microRNAs that suppress pro-apoptotic proteins, including thioredoxin-interacting protein (TXNIP) [79]. The upregulation of TXNIP results in the activation of the inflammasome and caspase-1-dependent pro-death pathway [79]. BH3-only proteins (Bid, Bim, Noxa, and Puma) are also affected by these miRNAs and are upregulated [79]. They inactivate mitochondrial-protecting proteins, such as Bcl-2 and Bcl-XL, and activate pro-apoptotic proteins, such as Bax and Bak, that permeabilize the outer mitochondrial membrane [79]. Another signaling pathway involved is the apoptosis-signal-regulating kinase 1 (ASK1)-c-Jun NH2-terminal kinase (JNK) axis, which activates Bim and inhibits Bcl-2 [48,79]. Sustained PERK activation upregulates the transcription factor C/EBP homologous protein (CHOP), which suppresses the expression of anti-apoptotic Bcl-2 to promote cell death [78,79] (See Figure 1). In the disc field, the accumulation of AGEs is reported to induce ER stress through dysregulated Ca^2+^ homeostasis in aged and degenerated discs, especially in diabetic patients [22].

### 2.2. Necroptosis

**Figure 2:**
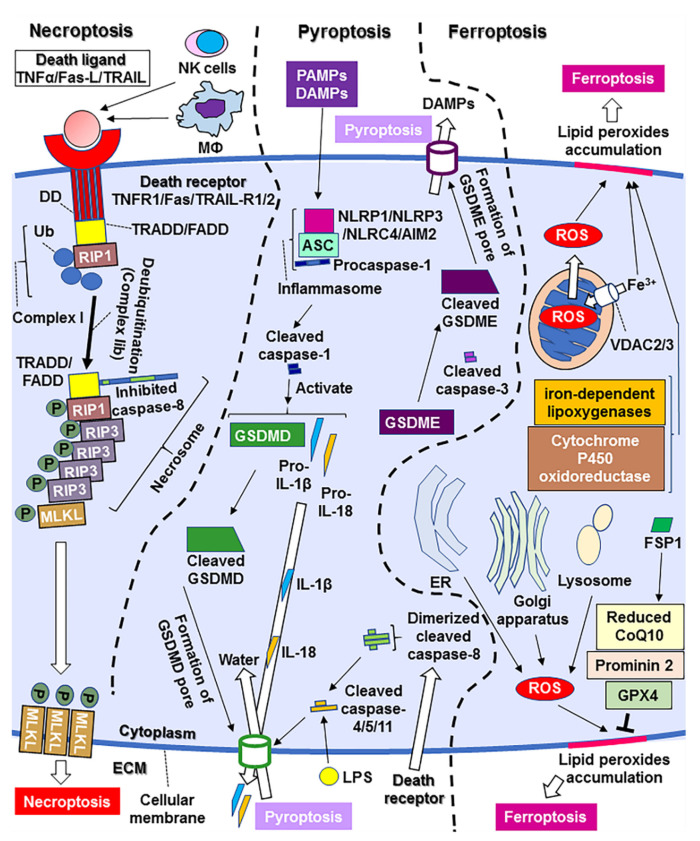
A scheme of necroptotic, pyroptotic, and ferroptotic signaling pathways. AIM2, absent in melanoma 2; ASC, apoptosis-associated speck-like protein; CoQ10, coenzyme Q10; DAMPs, damage-associated molecular patterns; DD, death domain; ECM, extracellular matrix; ER, endoplasmic reticulum; FADD, Fas-associated death domain; FSP, ferroptosis suppressor protein; GPX, glutathione peroxidase; GSDMD, gasdermin-D; GSDME, gasdermin-E; LPS, lipopolysaccharide; MLKL, mixed lineage kinase domain-like pseudokinase; NLRP, nucleotide-binding oligomerization domain (NOD)-like receptors family pyrin domain containing; NLRC, NOD-like receptors family caspase recruitment domain containing; PAMPs, pathogen-associated molecular patterns; PARP, poly (ADP-ribose) polymerase; PERK, pancreatic endoplasmic reticulum kinase; RIP, receptor-interacting proteins; ROS, reactive oxygen species; TNFR, TNF receptor; TRADD, TNFR-associated death domain; TRAIL, TNF-related apoptosis-inducing ligand; TRAILR, TRAIL receptor; TXNIP, thioredoxin-interacting protein; Ub, ubiquitination; XIAP, X linked inhibitors of apoptosis protein.

Necroptosis is a type of RCD characterized by necrosis [35]. When the cell is unable to enter apoptosis and death ligand binds to the receptor, pro-survival complex I forms [56], comprising TRADD/FADD, receptor-interacting proteins (RIP) 1, and several ubiquitin E3 ligases [54]. Deubiquitination of RIP1 results in the formation of complex IIa or IIb [35,56]. The turning point between subsequent apoptosis and necroptosis is determined by whether or not caspase-8 is inhibited [35]. When caspase-8 can become activated, complex IIa forms and activates caspase-8, triggering apoptosis [55]. However, complex IIb formation leads to necroptosis [35,55]. It involves the formation of a complex called necrosome consisting of TRADD/FADD, caspase-8, RIP1, and phosphorylated RIP3; RIP3 phosphorylates mixed lineage kinase domain-like pseudokinase (MLKL) [81], which oligomerizes and translocates to the cytoplasmic membrane from the cytoplasm to perforate pores and causes cell lysis, thereby resulting in necroptosis [35,54] (See Figure 2). RIP1/RIP3/MLKL-mediated necroptosis has been reported in compression-induced rat NP cell death [53] and in herniated human NP tissue and cells [81]. Therefore, necroptosis occurs in the vicinity of intervertebral discs.

### 2.3. Pyroptosis

Pyroptosis is an inflammatory form of RCD [58]. Pattern-recognition receptors (PRRs) include toll-like receptors (TLRs), nucleotide-binding oligomerization domain (NOD)-like receptors (NLRP1, NLRP3, and NLRC4), and absent in melanoma 2 (AIM2)-like receptors (ALRs); they constitute inflammasomes with apoptosis-associated speck-like protein (ASC) and pro-caspase-1 [60]. When PRRs recognize certain pathogen-associated molecular patterns induced by exogenous pathogens and damage-associated molecular patterns (DAMPs) derived from endogenous pathogens, inflammasomes lead to activation of caspase-1, and subsequent activation of interleukin (IL)-1β, IL-18, and gasdermin-D (GSDMD) [82]. The cleaved N-terminal fragment of GSDMD forms pores in the plasma membrane, leading to excess extracellular secretion of ILs and intracellular water flux, resulting in cell swelling and lysis [58,60]. Lipopolysaccharide (LPS) from gram-negative bacteria may also directly activate caspase-4/5/11 to cleave GSDMD and form plasma membrane pores [60]. In addition, GSDMD cleavage can be induced through the activation of caspase-4/5/11 by death receptor stimuli and caspase-8 activation [59,60]. The executor of apoptosis, caspase-3, has also been found to cleave gasdermin-E (GSDME), which can also perforate the plasma membrane [55]. In this case, both the stimulation of death receptors and the efflux of pro-apoptotic proteins from the mitochondria result in pyroptosis as well as apoptosis (See Figure 2). The relevance of pyroptosis in intervertebral discs was reported in the presence of *Propionibacterium acnes* infection [83], wherein the NLRP3 inflammasome was suggested to contribute to the induction of pyroptosis through TXNIP [83]. TXNIP inhibits thioredoxin and increases intracellular ROS production [83]. Another condition involving NLRP3-mediated pyroptosis is injury-induced disc degeneration [84]. Decreased levels of miR-410 were found in needle-punctured discs and miR-410 was implicated as a negative mediator of NP cell pyroptosis [84]. Although controversial, lactate accumulation may be responsible for NLRP3 inflammasome-mediated pyroptosis in degenerated disc tissue [85,86]. Injection of exogenous lactate has been shown to stimulate acid-sensing ion channel (ASIC) 1 and 3 to promote intracellular transportation of Ca^2+^, leading to an increase in intracellular ROS levels in NP cells [86]. Moreover, enhanced intracellular Ca^2+^ signaling has been suggested to be associated with the augmentation of ROS and NLRP3 inflammasome activation in blood cells [57]. Similar results have been demonstrated in NP cells wherein ASIC1 and ASIC3 induced NLRP3 inflammasome activation and pyroptosis in NP cells via upregulation of the ROS/NF-κB signaling pathway, followed by increased expression of inflammasome components [86].

### 2.4. Ferroptosis

Ferroptosis is a necrotic form of RCD characterized by lipid peroxidation and free iron-mediated Fenton reactions [87]. Cells undergoing ferroptosis accumulate lipid peroxides and are deficient in the defense system required to eliminate them, leading to their accumulation to lethal levels [36]. Ferroptotic cells exhibit necrotic morphology, small dysmorphic mitochondria with decreased crista, a condensed membrane, and a ruptured outer membrane [36,62,88]. It has been shown using the small molecule erastin that mitochondrial VDACs 2/3 open and take up iron, and iron-induced ROS generation results in elevated mitochondrial potential and oxidative stress-induced ferroptosis [89]. ER-derived oxidative stress, Golgi stress-related lipid peroxidation, and lysosomal dysfunction are attributed to ferroptosis [90,91]. Other factors include lipid peroxide generation by iron-dependent lipoxygenases and further propagation of peroxides by labile iron [36,92]. Cytochrome P450 oxidoreductase has also been reported to be a driver of lipid peroxidation during ferroptosis [36,93]. Glutathione peroxidase (GPX) 4 is a representative suppressor of ferroptosis; it prevents the accumulation of peroxidized lipids [36]. Ferritin contributes to a decrease in free iron which is supplied through VDAC 2/3 [36]. Prominin 2 sabotages polyunsaturated fatty acyl phospholipids that are crucial for iron-dependent lipid peroxidation [36]. Coenzyme Q10 (CoQ10) is a part of the mitochondrial electron transport chain; reduced CoQ10 can trap lipid peroxides and prevent ferroptosis outside the mitochondria [36,63]. Ferroptosis suppressor protein (FSP) 1 reduces CoQ10 and decreases lipid peroxides [63] (See Figure 2). The major implications of ferroptosis in disease are found in cancer as well as in the nervous system and digestive system disorders [61,62,94]. Ferroptosis has also been implicated in the vicinity of intervertebral discs [87]. The levels of GPX4 and ferritin heavy chain were lower in the degenerated disc tissues of a rat model of IVDD than in those of healthy rats [87]. In addition, ferroptosis may also be attributed to injury-induced disc degeneration because the degenerative processes have been shown to be attenuated by deferoxamine, an inhibitor of ferroptosis, in a rat model of IVDD [87].

## 3. Targets to Regulate PRCD to Prevent Disc Degeneration

In this section, we review previously studied therapeutic targets to prevent cell loss due to PRCD and the consequent IVDD, focusing on siRNA and ncRNAs.

### 3.1. Small Interfering RNA (siRNA)

Small interfering RNAs (siRNAs) are often targeted for inhibiting certain genes at the transcriptional level [95,96]. The executor of apoptosis, caspase-3 is an effective target to inhibit cell death [70]. Previous studies have shown caspase-3 siRNA to be effective for not only inhibiting apoptosis in cultured NP cells, but also as a therapeutic reagent to prevent injury-induced or biomechanical overload-induced IVDD [68,70,74]. Another study showed the modulation of CHOP, a product of ER stress, by CHOP-siRNA, as well as a significant reduction in the rate of apoptosis, ROS level, lysosome activity, and expression of PARP, Caspase-12, Caspase-3, LC3, Beclin-1, and CHOP, in an in vitro cyclic deformation stress model [33]. Yes-associated protein 1 (YAP1), a transcriptional co-activator and negative regulator of the Hippo pathway, regulates cell proliferation, contact inhibition, and tissue size [97]. Chen et al. reported that YAP1 is activated by IL-6 through tyrosine phosphorylation, and YAP1 knockdown by siRNA increased Sox-9, type II collagen, and aggrecan expression in IL-6-treated NP cells [97]. Notably, IL-6 was shown to enhance YAP1-β-catenin interaction and nuclear accumulation, and β-catenin knockdown by siRNA has been shown to block IL-6 treatment or YAP1 overexpression induced degenerative consequences [97]. The YAP1 inhibitor verteporfin was used in an injury-induced disc degeneration model and partially rescued type II collagen and inhibited MMP-13 [97]. TXNIP, an endogenous inhibitor of thioredoxin, produces excessive ROS and causes cellular oxidative stress; high levels of TXNIP accompanied by inflammasome activation have been found in degenerated discs [83]. In an in vitro NP cell co-culture system with *P. acnes*, targeting of the TXNIP/NLRP3 signaling pathway using the NLRP3 inflammasome inhibitor MCC950 and TXNIP-siRNA led to reduced secretion of mature IL-1β and IL-18 [83]. The efficacy of MCC950 was further established in vivo in a rabbit model of IVDD with *P. acnes* infection, which showed an alleviatory effect of MCC950 on the degenerative process [83]. Lactate accumulation is an important factor in disc degeneration [86]. In vitro, lactate exposure to NP cells enhanced pyroptosis, and NLRP3-siRNA or ASIC inhibitors successfully blocked lactate-induced NLRP3 inflammasome activation [86]. As explained in the previous section, extracellular lactate controls the levels of intercellular ROS through ASIC1 and ASIC3 [86]. ROS activates the NF-κB signaling pathway and upregulates the expression of NLRP3 inflammasome components and IL-1β release [86]; therefore, NLRP3-siRNA and ASIC inhibitor treatment inhibit pyroptosis. A comparative analysis of the miRNA profiles of NP cells collected from healthy subjects and those with degenerative discs revealed that bone morphogenetic protein (BMP) 2 is a target of miR-129-5p, and downregulation of miR-129-5p expression is a risk factor for IVDD as it promotes apoptosis in NP cells [98]. NP cells treated with miR-129-5p mimics or BMP2-siRNA exhibited improved viability and lower apoptosis rates than the control groups [98]. The Fas death receptor is a therapeutic target for inhibiting apoptosis via the extrinsic pathway. A previous study has shown a reduction in apoptosis and improved annulus fibrosus cell proliferation with Fas-siRNA in a serum starvation model [99]. Bim, a member of the BH3 subfamily of the Bcl-2 family is also targeted for inhibiting apoptosis. Bim has been found to be a direct target of miR-25-3p, which was downregulated in NP cells in degenerative discs [100]. miR-25-3p inhibition decreased NP cell proliferation and induced cell apoptosis but Bim-siRNA inhibited apoptosis [100]. The application of Bim-siRNA may also be an attractive treatment for preventing apoptosis and subsequent IVDD. Therefore, targeting various pro-apoptotic molecules through silencing via the siRNA approach is an effective method to prevent IVDD.

### 3.2. ncRNAs

NcRNAs are a group of RNAs that regulate mRNAs, including microRNAs, long ncRNAs (lncRNAs), and circular RNAs (circRNAs) [101]. Intriguingly, evidence has shown that ncRNAs play an important role in the occurrence of disc degeneration.

MiR-486-5p has been shown to be significantly lower in degenerated discs than in controls [102]. Another study revealed that miR-486-5p directly targets fork head box protein O1 (FOXO1), which regulates the expression of inflammatory cytokines and plays a vital role in apoptosis [102]. Thus, the upregulation of miR-486-5p may be a strategy for inhibiting cell death and disc degeneration. MiR-141 is another miRNA that is known to be agonistic to NP cell apoptosis [103]. MiR-141 was found to deplete SIRT1, a negative regulator of the NF-κB pathway, and knocking out miR-141 attenuated spontaneous and surgically induced IVDD [103]. MiR-141 may therefore be a potential target for preventing disc degeneration. MiR-138-5p [30,104] and miR-34a-5p [77] also negatively affect SIRT1. Another miRNA, miR-27a overexpression induces apoptosis of human degenerated NP cells by silencing phosphatidylinositol 3-kinase (PI3K) [30]. The PI3K/protein kinase B (Akt) pathway determines cell fate by modulating cell proliferation, apoptosis, autophagy, and differentiation [30]. Therefore, miR-27a inhibition may be an attractive strategy for preventing cell death in degenerative discs [30]. MiR-494 has also been shown to promote NP cell apoptosis [105,106] by negatively regulating JunD and subsequently promoting the secretion of pro-apoptotic proteins from the mitochondria [105,106], making it a candidate therapeutic target for the prevention of degenerative cell loss. In contrast, the expression of miR-155 has been found to be low in degenerative human NP cells [105,107], whereas its overexpression resulted in the downregulation of FADD and caspase-3 and subsequent decrease in Fas-mediated apoptosis in human NP cells [105,107]. MiR-532 was found to be upregulated in degenerated discs, and treatment with miR-532 mimics increased NP cell apoptosis [108]. MiR-532 was shown to downregulate Wnt/β-catenin signaling via targeting Bcl-9, therefore leading to NP cell apoptosis [108]. MiR-185 binds to and negatively regulates Galectin-3, which is highly expressed in NP cells of degenerated discs [109]. As a simulation of degenerated disc conditions, the effects of miR-185 inhibition were assessed and found to increase Galectin-3, pro-autophagy factors LC3 and Beclin-1, pro-apoptosis factors caspase-3 and Bax, as well as the Wnt/β-catenin signaling pathway [109]. Notably, miR-185 agomir injection alleviated the degenerative processes induced in the instability model [109]. MiR-210 is known to regulate apoptosis and its expression levels were found to be lower in patients with degenerative discs than in those with non-degenerate discs [110]. When NP cells treated with FasL were additionally treated with pre-miR-210, the number of apoptotic cells significantly decreased [110], suggesting a protective role of miR-210 against apoptosis through the extrinsic pathway. MiR-410 is known to be an important negative mediator of pyroptosis and acts by suppressing the NLRP3/caspase-1 pathway [84]. Therefore, miR-410 may be a potential target for regulating pyroptosis in NP cells. MiR-222 is also upregulated in human degenerative disc tissues [111]. In vitro, apoptosis was promoted and inhibited and the production of TNF-α, IL-1β, IL-6, TLR4, p-IκΒα, and p-p65 were upregulated and downregulated by miR-222 mimics and miR-222 inhibitors, respectively [111]. Moreover, tissue inhibitor of metalloproteinase 3 (TIMP3), a suppressor of matrix degradation was elucidated to be a direct target of miR-222 [111]. Therefore, miR-222 inhibitor may be ideal for suppressing cell death and preserving extracellular matrix integrity, including that of intervertebral discs. Besides the aforementioned mRNAs, several other miRNAs, including miR-21 [112], miR-499a-5p [113], miR-125a [114], miR-145 [115], and miR-573 [116] are anti-apoptotic, and miRNAs including miR-30d [117], miR-222-3p [118], miR-15a [119], miR-143 [120], miR-106a-5p [121], and miR-221 [122,123], are also pro-apoptotic. As mentioned in the previous section, miR-129-5p prevents apoptosis of NP cells by targeting BMP2 [98], and miR-25-3p protects NP cells from apoptosis, whereas Bim siRNA can be an option to disinhibit miR-25-3p [100]. In conclusion, agomirs and antagomirs of miRNAs whose low and high expression, respectively, is associated with disc degeneration can be used for their upregulation and downregulation, respectively, to prevent the death of NP cells and subsequentIVDD.

The lncRNA RP11-296A18.3 has been implicated in excess NP cell apoptosis; it has been found to be upregulated in degenerative discs [124,125]. HLA complex group 18 (HCG18) is another lncRNA that has been found to be upregulated in NP tissues from herniated or bulging discs [124,126]; HCG18 acts as an endogenous sponge for miR-146a-5p to inhibit cell proliferation, promote apoptosis, and enhance the release of chemoattractants for macrophages in NP cells [124,126]. Such upregulated lncRNAs can be targeted for inhibiting disc degeneration. In contrast, metastasis-associated lung adenocarcinoma transcript 1 (MALAT1 alias, NEAT2) is a lncRNA that is downregulated in NP cells isolated from degenerative discs [124,127]. MALAT1 has been shown to inhibit caspase-3 activity and secretion of IL-1 and IL-6 [124,127]; hence, it can be therapeutically enhanced to prevent disc degeneration. The lncRNA prostate androgen-regulated transcript 1 (PART1) has been reported to promote disc degeneration by downregulating the miR-93/MMP2 pathway in NP cells [128,129]. A study showed that PART1 knockdown enhances cell viability, reduces cell apoptosis, inhibits inflammatory factor secretion, and promotes matrix degradation in LPS-stimulated NP cells in vitro [128]. Further studies revealed that PART1 sponges miR-190a-3p and downregulates its expression [128]. Therefore, PART1 inhibition or miR-190a-3p overexpression may be an option to prevent apoptosis. TUG1 is a pro-apoptotic lncRNA that upregulates the levels of Bax and caspase-3 in the Wnt1/β-catenin pathway and downregulates the levels of Bcl-2 [122,130]. GAS5 and lncPolE are pro-apoptotic lncRNAs that are overexpressed in degenerative discs [122,131,132]. GAS5 sequesters to miR-155 and lncPolE downregulates PolE to promote apoptosis [122,131,132]. Therefore, lncRNA can be inhibited or overexpressed to prevent NC cell apoptosis and thereby, IVDD.

CircVMA21, circRNA derived from the vacuolar ATPase assembly factor (*VMA21*) gene targets miR-200c, and circVMA21 indirectly affects XIAP through miR-200c [133]. CircVMA21 is known to act as a sponge for miR-200c, which exhibits tumor suppressive and apoptosis-inducing behavior [133]. MiR-200c inhibits XIAP and reduces NP cell viability, and direct injection of circVMA21 in intervertebral discs was found to alleviate NP cell apoptosis and IVDD [133]. CircRNA involved in compression-induced damage of NP cells (circRNA-CIDN) have been found to be downregulated in compression-treated human NP cells and circRNA-CIDN overexpression inhibited compression-induced apoptosis in NP cells [77]. Further investigation revealed that circRNA-CIDN acts as a sponge for miR-34a-5p, which plays a detrimental role in compression-induced damage of NP cells by repressing SIRT1 [77]. In contrast, circRNA_104670 acts as a sponge for miR-17-3p, abrogating its protective role in NP cell apoptosis [77,134].

## 4. Future Directions and Conclusions

IVDD is a formidable health problem that is associated with back pain [2], spinal segment instability [135,136], and secondary neurological deficits [137]. One of the intractable problems associated with IVDD is the lack of fundamental treatment. Numerous studies on the treatment of IVDD have been performed worldwide, and the major principle of all therapeutic strategies is the prevention of cell loss due to excessive PRCD by targeting mRNAs [33,70], ncRNAs [77,100], hormones [46,138], proteins related to PRCD [139,140], autophagy [141,142], and cellular homeostasis [82,143] or utilizing vitamins [73,144], natural compounds [145,146], growth factors [147,148], synthetic drugs [31,76], and traditional Chinese medicine [149,150]. In this study, we summarized the molecular pathways of PRCD and the molecular targets that can be modulated at the mRNA level to suppress PCD and other types of RCD.

However, there exist several difficulties in applying these RNA interventions for preventing IVDD in clinical settings, and they must be addressed. Early-stage interventions cannot be highly invasive because patients with early to middle stage IVDD may not exhibit severe symptoms or disability. However, to prevent the progression of IVDD, interventions during the window of opportunity should be important. New approaches, such as novel drug delivery systems, may need to be explored to realize early-stage treatments for IVDD. Further research is necessary to improve the quality of life of patients.
